# Regulation of secretory pathway kinase or kinase-like proteins in human cancers

**DOI:** 10.3389/fimmu.2023.942849

**Published:** 2023-02-07

**Authors:** Shaonan Du, Chen Zhu, Xiaolin Ren, Xin Chen, Xiao Cui, Shu Guan

**Affiliations:** ^1^ Department of Neurosurgery, Shengjing Hospital of China Medical University, Shenyang, China; ^2^ Department of Neurosurgery, The First Hospital of China Medical University, Shenyang, China; ^3^ Department of Neurosurgery, Shenyang Red Cross Hospital, Shenyang, China; ^4^ Department of Surgical Oncology and Breast Surgery, The First Hospital of China Medical University, Shenyang, China

**Keywords:** secretory pathway, kinase, SPKKPs, tumor, microenvironment

## Abstract

Secretory pathway kinase or kinase-like proteins (SPKKPs) are effective in the lumen of the endoplasmic reticulum (ER), Golgi apparatus (GA), and extracellular space. These proteins are involved in secretory signaling pathways and are distinctive from typical protein kinases. Various reports have shown that SPKKPs regulate the tumorigenesis and progression of human cancer *via* the phosphorylation of various substrates, which is essential in physiological and pathological processes. Emerging evidence has revealed that the expression of SPKKPs in human cancers is regulated by multiple factors. This review summarizes the current understanding of the contribution of SPKKPs in tumorigenesis and the progression of immunity. With the epidemic trend of immunotherapy, targeting SPKKPs may be a novel approach to anticancer therapy. This study briefly discusses the recent advances regarding SPKKPs.

## 1 Introduction

Kinases are evolutionarily conserved enzymes for the regulation of many cellular processes by transferring phosphate molecules from ATP to target substrates (1, 2). The human kinome encompasses more than 500 kinases and acts as molecular activators and signal transducers. Solid tumors, among other disorders, present defective and abnormal activation of kinases (3). It has been identified in recent years that a group of proteins, the “secretory pathway kinase or kinase-like proteins” (SPKKPs), is explicitly observed in the lumen of the endoplasmic reticulum (ER), Golgi apparatus (GA), and extracellular space (4–6). The mutation of SPKKPs will result in disease (7), demonstrating the biological importance of phosphorylation in the secretory pathway.

**Figure 1:**
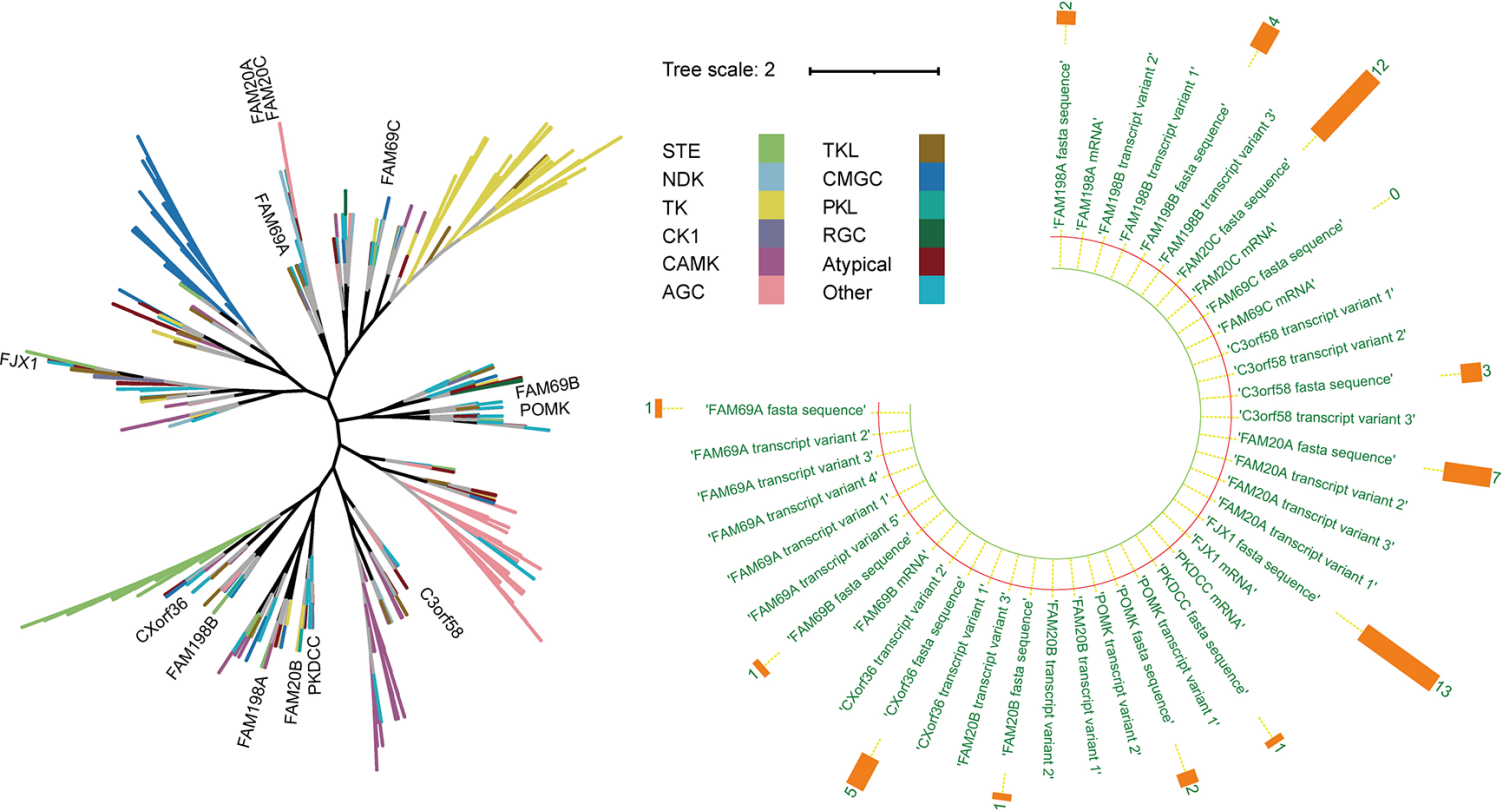
Representation of the evolutionary relation analysis of the secretory pathway kinase or kinase-like proteins in human kinome. Left: The black branches represent the development process of the phylogenetic tree of all kinases (branches of different colors represent different kinase groups). Right: The orange bar chart represents the number of disease literature related to the corresponding genes.

**Figure 2:**
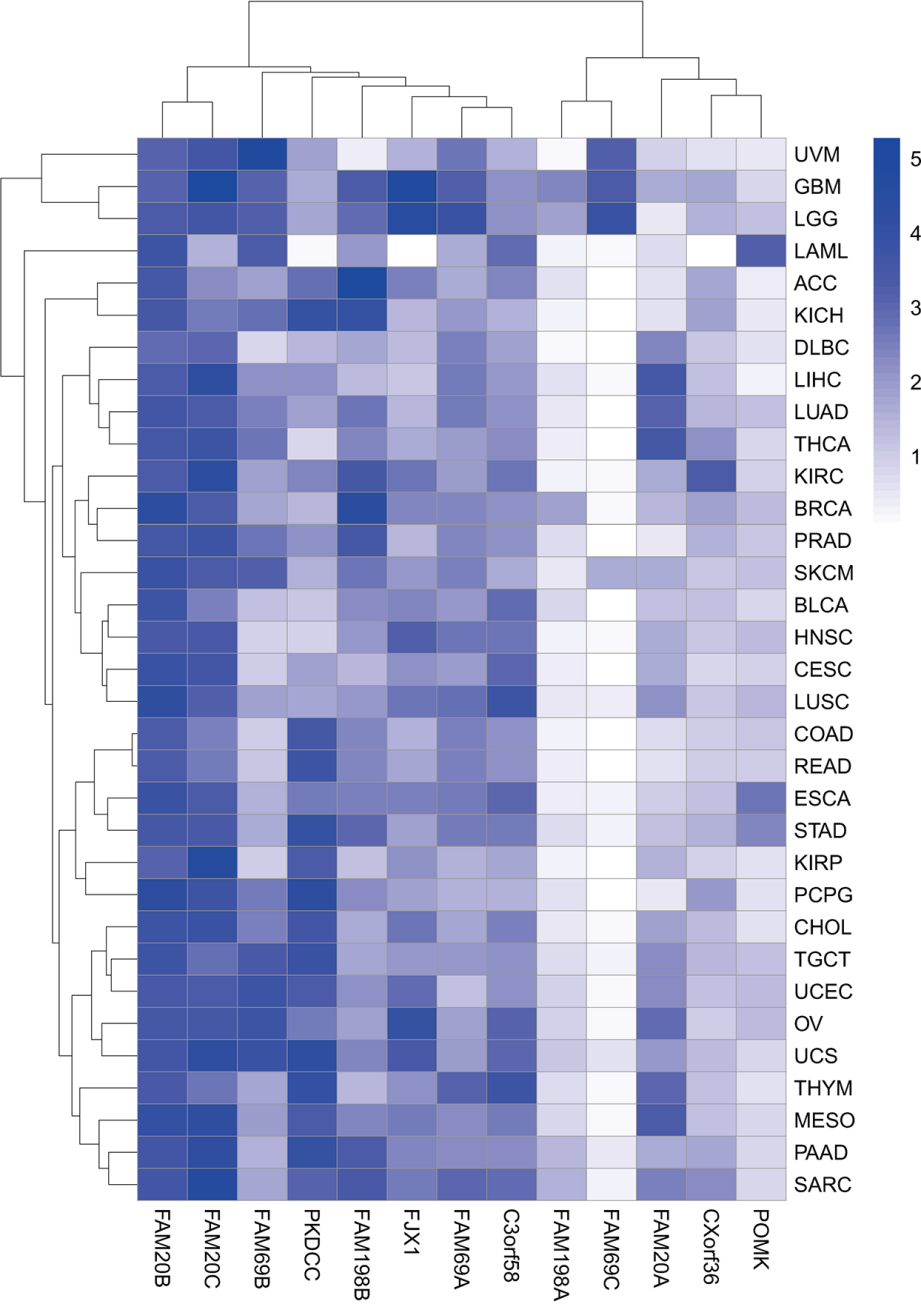
Heatmap of gene expression in pan-cancer. Heatmap showing the association of 13 genes encoding secretory pathway kinases or kinase-like proteins with indicated cancer types in the TCGA pan-cancer RNA sequencing dataset downloaded from UCSC Xena (https://xena.ucsc.edu). The color code represents the expression level of the 13 genes, with the most significant high expression in dark blue.

This group of kinases can phosphorylate several substrates that are crucial to pathological processes, such as tumor growth and metastasis, and act on secretory molecules as glycan kinases (3, 8–11). In addition, some kinase-like proteins are widely present in the enzyme family, such as FAM20C-related kinases, PKDCC-related kinases (8), and POMK (9), and all are actively involved in a series of cell functions (8). The evolution of multiple secreted kinases appears to occur as independent events rather than diverging from primordial secreted kinases, as shown by the fact that this group of genes is more evolutionarily distant from one another than canonical cytoplasmic kinases (**Figure 1**) (12). Nonetheless, the functions of these SPKKPs in cancers remain poorly defined. Bioinformatic analyses unveiled that most of these proteins are presented in a tumor-promoting phenotype (**Figure 2**). This review specifies the kinases involved in the secretory pathway and emphasizes the importance of the kinases in human cancer.

## 2 Secretory pathway

Information transmission between cells is the fundamental guarantee of cell growth, differentiation, development, proliferation, apoptosis, and other life activities in organisms. Protein secretion is an important way of information transmission between cells. More than 30% of human genes are coding proteins for the extracellular environment, cell membrane, or secretory pathway components (13). The intracellular secretory pathway is an essential physiological process and consists of the ER, GA, and associated vesicles. These components fold, modify, and transport proteins to the lysosome, plasma membrane, and extracellular space (14). The Nobel prize in Physiology or Medicine in 1999 was awarded to Dr. Günter Blobel for understanding the mechanism of secretory pathway entry. The newly synthesized proteins enter the secretory pathway through signal recognition particles. These particles are folded and modified in the ER, and the proteins are involved in the transportation and localization in the secretory pathway, such as transport through vesicles between the ER and Golgi, localization in the ER, modification in the GA, and transport through the GA. Then, the proteins leave the GA and target the post-Golgi locations (including lysosome, plasma membrane, and extracellular space) (15).

Secretory proteins include extracellular matrix proteins and autocrine/paracrine proteins (16), which are involved in various physiological and pathophysiological processes, including immune defense, cell signal transduction, tumor microenvironment modification, angiogenesis, invasion, and metastasis. All proteins targeting the extracellular space pass through the ER, the starting point of the metabolic pathway, to reach the cell surface. A cancer secretome is a subproteome of the collection of all proteins secreted or escaped from cancer cells.

Kinases or kinase-like proteins in the secretory pathway seem to play critical roles in the tumor microenvironment. PKDCC (also named VLK) and FAM20C are the first two identified secreted kinases discovered and involved in the phosphorylation of cell surface proteins (12, 17). Other secretory kinases are subsequently identified, such as FJX1 and FAM20A. This group of proteins is crucial in promoting tumor processes, including cell invasion and colony formation (18), as they are part of the immune environment. The kinase secretomes are valuable sources of cancer biomarkers or therapeutic targets due to their interaction with several growth factors, receptors, adhesion molecules, and extracellular matrix components.

## 3 Physiological processes and pathological results of the secretory pathway

Secretory and transmembrane proteins are produced in the ER and transported to the Golgi compartment for further maturation. Various mechanisms are evolved by eukaryotic cells to preserve the efficiency and fidelity of protein production (19). The ER and Golgi guarantee that only the correctly folded proteins should be released through the secretory pathway and those improperly folded should be degraded. However, in some instances, the loss of enzymes or proteins causes the dysfunction of the ER and GA and affects the secretory pathway, contributing to various human diseases.

### 3.1 ER protein folding and ER stress

Approximately one-third of the proteomes in eukaryotes are transported through the secretory pathway, where nascent proteins are the first target in the ER. Fundamental modifications necessary for their maturation are then completed to ensure that the protein is appropriately structured before being released to the predetermined cell compartment (20, 21). ER protein folding, an error-prone process, may result in multiple conformations in the process of folding (22). Misfolded proteins accumulated in the ER under environmental stresses will promote tumorigenesis (23). The protein folding process is coordinated with the quality control system, and the improperness of protein folding results in ER stress (24). Mammalian ER stress responds to misfolded protein damage *via* multiple mechanisms, including weakening of translation, promotion of chaperone protein expression, enhancement of protein degradation components, and induction of apoptosis (25). On the other hand, chronic ER stress triggered by tumor microenvironment factors can interfere with protein folding (26).

The protein disulfide isomerase (PDI) family is a prominent participant in protein folding (27). Recent studies have found that FAM20C phosphorylates Ser357 in PDI and induces an open conformation, changing the state from “foldase” to “holdase.” This is essential in the prevention of protein misfolding in the ER. Most FAM20C is retained in the ER and forms a condensate with PDI, which leads to a higher local concentration of FAM20C and catalyzes phosphorylation during ER stress (28). In addition, ER stress also increases ER ATP levels through AXER (an ATP/ADP exchanger on the endoplasmic reticulum membrane), which may also promote FAM20C-catalyzed phosphorylation (29). FAM20C phosphorylates PDI on Ser357 upon ER stress and promotes the activity of PDI to maintain ER proteostasis in acute liver damage (28). The FAM20C–PDI axis contributes to the post-translational response for ER protein homeostasis maintenance and is important in cell protection from cell death induced by ER stress (28, 30).

### 3.2 Post-translation modification in the ER

Both N-glycosylation and O-mannosylation are critical for protein maturation. N-linked glycosylation sites, generally composed of asparagine, non-proline amino acids, and serine/threonine sequences, are the attachment sites of high mannose oligosaccharides (31). O-mannosylation is effective on the Ser and Thr side chains to improve protein stability and solubility (32). POMK (protein-O-mannose kinase) encodes protein-O-mannose kinase, which is essential in extracellular matrix composition and involved in the proper glycosylation and function of the dystroglycan complex (33, 34).

### 3.3 Unfolded protein response

Growing evidence supports the critical role of unfolded protein response (UPR) in tumor establishment, progression, metastasis, and chemoresistance, as well as its involvement in acquiring other hallmarks of cancer (35). The activation of the UPR can support and promote the survival of tumor cells by improving the folding ability of ER chaperones and promoting the ER-related degradation (ERAD) pathway (28). However, the functions of UPR activation also include tumor growth inhibition, especially when ER stress is intense and persistent in cancer cells. In this case, the UPR is not able to solve the protein folding defect but instead induces cancer cell death (28). Therefore, it is necessary to accurately and timely regulate ER protein homeostasis and UPR signaling in the physiological state.

The UPR is a dynamic signaling network composed of three distinct arms in mammals, i.e., the ER transmembrane sensors IRE1a, PERK, and ATF6 (36, 37). The negative regulation of the IRE1a signal by the FAM20C–PDI axis is physiologically relevant since the terminal UPR contributes to various diseases, including diabetes, cancer, and neurodegeneration (30). Recent evidence has shown that the IRE1a branch of UPR is sensitized by FAM20C depletion under ER stress (28). Phosphorylation of FAM20C reduces the XBP1 mRNA splicing level in thapsigargin-treated HepG2 cells, and XBP1 can coordinate many changes in cell structure and function, leading to the characteristic phenotype of secretory cells (38). Caveolin-1, which is closely related to FAM198A and retained in the ER (39–42), regulates melanoma by modulating the secretory pathway in a manner depending on the inhibition of the UPR (43). PKDCC regulates the transport of ERP29, RP44, BiP, and PDIA3 by phosphorylating transport receptors. These proteins are components of ER proteostasis and play an important role in UPR-induced apoptosis (44). Therefore, FAM20C, FAM198A, and PKDCC are essential in regulating ER proteostasis and UPR signaling.

### 3.4 Golgi apparatus stress and mitochondrial stress

All organelles related to protein quality control, including the GA and mitochondria, are sensitive to the accumulation of unfolded proteins and have different strategies to mitigate their accumulation (45, 46). There are few studies on the molecular pathways concerned with the stress response of these organelles. The GA is mainly used to coordinate the post-translational modification of proteins and transport proteins and lipids to their destination. Kinoshita et al. reported that ectopically expressed PKDCC co-localized with Golgi markers (47). Mattia R. Bordoli showed that PKDCC either is presented and active in the ER or phosphorylates ER-resident proteins in the GA during the cycling between these compartments (12).

GA is the main sorting hub of the intracellular protein secretory pathway. Sorting is done in the trans-Golgi network (TGN) (48–50). Cab45 can regulate the sorting and secretion of newly synthesized proteins through a Ca2^+^-mediated process in the TGN (51). FAM20C phosphorylates Cab45 clients (52, 53) on five distinct residues (S99, S142, T131, T193, and S349), thereby decreasing Cab45 retention in the TGN (51). Another secretory pathway kinase C3orf58 (also named DIPK2A) enhances autophagosome–lysosome fusion by binding VAMP7B, contributes to the autophagic degradation of mitochondria proteins, and alleviates apoptosis (54).

## 4 The role of the secretory pathway kinases or kinase-like proteins in human cancer

The research on SPKKPs has never stopped. With the development of science and technology, more and more literature is demonstrating the expression and effect of SPKKPs in different tumors. We have sorted out some literature and the summary is shown in **Table S1**.

### 4.1 FAM20C-related kinases

#### 4.1.1 Tumor promoter: FAM20C

FAM20C is a critical component of the secretory pathway-related kinase (8). In 2007, Simpson et al. discovered that FAM20C mutations in humans can lead to Raine syndrome (55), an autosomal recessive osteosclerotic bone dysplasia first reported in 1989 (56). Patients generally develop generalized osteosclerosis, ectopic calcification, and skull and facial features, i.e., craniosynostosis, exophthalmos, microcephaly, proptosis, depressed nasal bridge, and midface hypoplasia. In 2012, researchers found that FAM20C regulates FGF23 (57, 58), which is secreted by osteoblasts and acts on the kidney to regulate phosphate resorption (59). FAM20C causes hereditary hypophosphatemic rickets by activating the missense mutation of FGF23 (60). In 2016, Vincent S. Tagliabracci et al. found that the phosphorylation of soluble FAM20C-dependent secreted proteins promotes breast cancer cell migration (8). Our study published in 2020 showed that several types of solid tumors, i.e., breast, cervical, colorectal, esophageal, and pancreatic cancers, and especially glioma, had an elevated FAM20C (18). This result has been recognized by peers (61, 62). It was also found that increased FAM20C expression is related to decreased cisplatin sensitivity in testicular cancer (63).

**Figure 3:**
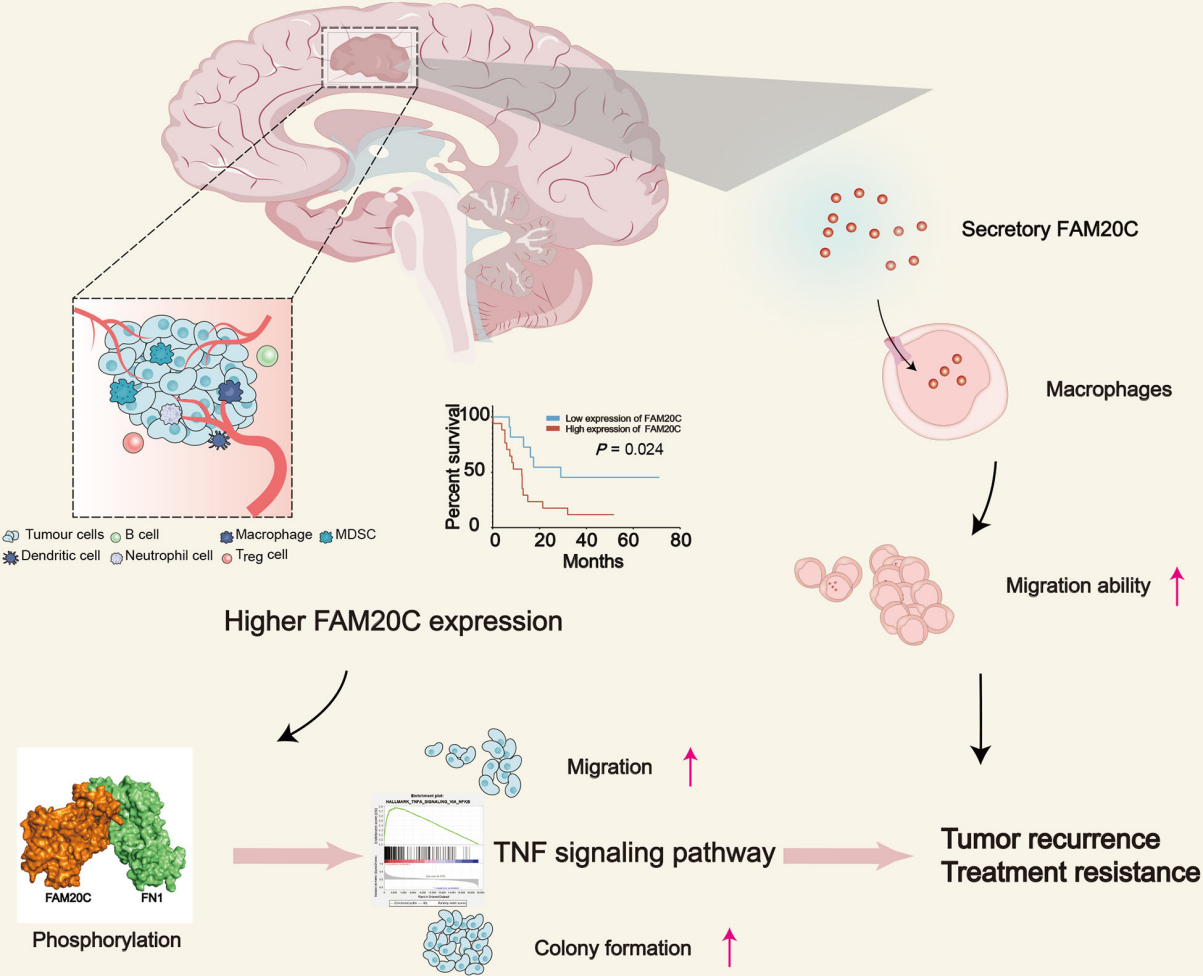
The schematic diagram depicts the role of FAM20C in promoting GBM progression. GBM with high FAM20C expression is associated with poor prognosis, and FAM20C promotes the migration and clone formation of tumor cells by binding with FN1 and activating the TNF signaling pathway. Also, FAM20C secreted by tumor cells promotes the migration of tumor-associated macrophages, which plays an important role in the development of tumors, modulation of neoangiogenesis, immune suppression, and metastasis. This may lead to tumor recurrence and treatment resistance.

FAM20C phosphorylates secretory proteins on s-x-e/PS motifs (64, 65). The first 22 resides of FAM20C encode a predicted signal sequence with FAM20C as the target to the secretory pathway where its substrates are encountered (66). Vincent S. Tagliabracci et al. reported that more than 100 secreted phosphoproteins were identified as genuine FAM20C substrates using CRISPR/Cas9 genome editing, mass spectrometry, and biochemistry (8). FAM20C substrates are implicated in tumor progression and immunotherapy. The deletion of the *PCSK9* gene, one of the FAM20C substrates, substantially attenuates cancer cell growth in mice. This attenuation depends on cytotoxic T cells and boosts the response of tumors to immune checkpoint therapy targeted at the checkpoint protein PD1 through a mechanism independent of PCSK9’s cholesterol-regulating functions (67). Inhibition of IGFBP3, another FAM20C substrate and a downstream effector of the YTHDF2-MYC axis in glioblastoma stem cells (GSCs), decreases GSC viability without affecting NSCs and impairs glioblastoma growth *in vivo* (68). The proprotein convertase 7 (PCSK7) binds to the liver-derived apolipoprotein A-V (apoA-V), non-enzymatically enhancing its degradation. This process starts in the ER and implicates acidic lysosomes in hepatocyte-derived human HuH7 cells (69). A low-frequency coding variant of the PCSK7 (R504H; SNP rs142953140) mutant enhances PCSK7 Ser505 phosphorylation at the exposed Ser-X-Glu507 motif recognized by the secretory pathway kinase FAM20C in a structural manner (8). Ser phosphorylation inhibits PCSK7-induced degradation of apoA-V (69). FN1 is a key substrate interacting with FAM20C (18) and was also reported to promote migration and invasion of glioma cells (70, 71). OPN phosphorylation by FAM20C decreases OPN secretion and reduces osteoclast differentiation and bone metastasis. In contrast, FAM20C in breast cancer cells promotes bone metastasis by facilitating the phosphorylation and secretion of BMP4, which in turn enhances osteoclastogenesis (62). Surprisingly, there are few overlapping substrates among the different cell lines. This indicates that individual cell populations have different microenvironments of secreted proteins. Therefore, the loss of multiple FAM20C substrate phosphorylation contributes to cell motility defects, and the inhibition of FAM20C may be a viable therapeutic approach to prevent tumor growth and metastasis (**Figure 3**).

Our research team recently discovered the role of FAM20C in glioma progression to promote the migration of THP1 cells at low concentrations (18). Xinpeng Liu et al. reported a marked relationship between FAM20C and CD4^+^ T cells, macrophages, neutrophils, and DC infiltration in bladder urothelial carcinoma, brain lower-grade glioma, and stomach adenocarcinoma through pan-cancer analysis. The authors found that FAM20C potentially regulates tumor-associated macrophage polarization by inducing Treg cell activation and T-cell exhaustion. Furthermore, a strong correlation was discovered between the FAM20C expression and T helper cell markers (Th1, Th2, Th9, Tfh, and Th17). The results suggested that FAM20C may influence both the extent of immune infiltration and the activation of diverse immune cells by regulating T-cell activation (72). In conclusion, secreted FAM20C plays an important role in the immune microenvironment.

#### 4.1.2 FAM20A

FAM20A is a pseudokinase due to its lack of key active site residues of kinase activity (17, 73). It is reported in some literature that FAM20A participates in the regulation of FAM20C activity by crystal structure analysis (9, 74), and mutations in FAM20A also cause amelogenesis imperfecta, enamel–renal syndrome, and gingival fibromatosis syndrome (75–77).

Ya Hu et al. found that *FAM20A* is one of the potential cancer-driver gene mutations, which is identified in more than one parathyroid carcinoma frozen sample by full-length genome sequencing (78). Li et al. identified the role of FAM20A in the regulation of the balance between epithelial proliferation and differentiation (79). The latest research showed that FAM20A can predict the early lymphatic metastasis of thyroid cancer (80). Wu et al. identified the role of FAM20A in IFNa treatment and its potential as a prognostic marker in hepatocellular carcinoma (HCC) (81). Genome-wide analysis revealed the differential expression of FAM20A in HCC based on serum alpha-fetoprotein levels and verified that FAM20A proteins directly interact with alpha-fetoprotein. FAM20A is associated with poor survival of HCC. Further experimental investigation on FAM20A should be performed to evaluate its potential as a diagnostic biomarker for the early diagnosis of HCC in a high-risk population (82). Interestingly, FN1 was co-screened with FAM20A in the above two articles, and FN1 was also found to act as a substrate of FAM20C and is essential in the development of glioma (18). FAM20A can form a complex and activates FAM20C allosterically to promote the phosphorylation of FAM20C targets (9). Therefore, the tumor promotion effect of FAM20A may be accomplished through the activation of FAM20C. A recent study showed that FAM20A can be used as a prospective biomarker for the diagnosis and biomaterial drug therapy of cartilage lesions (83). FAM20A is involved in cartilage regeneration and mainly related to immune response and cell chemotaxis, thus can be used as the final nanomedicine immunotherapy in regenerative medicine (83).

#### 4.1.3 FAM20B

The kinase family with the sequence similarity member of 20-B (FAM20B) is a xylose kinase essential for phosphorylating the xylose in the common linkage region of glycosaminoglycans (GAGs) (11). The latest research showed that FAM20B-catalyzed GAGs modulate the commitment of dental epithelial stem/progenitor cells to control the tooth number in mice *via* a mechanism including the restriction of FGFR2b signaling at the initial stage of tooth development (84). It is also important in the development of annulus fibrosus *via* the regulation of multiple signaling pathways, such as the TGF-β and MAPK pathways (85). Moreover, FAM20B is one of the chondroitin sulfate chains modifying enzymes and has weak positive staining in osteoarthritis and Kashin–Beck disease (86). Inactivated FAM20B in epiphyseal cartilage leads to chondrosarcoma in the knee joint and remarkable defects of postnatal ossification in the long bones; also, GAG deletion and chondrocyte overproliferation are observed in this process (11, 87). Mechanistic analyses revealed that FAM20B-deficient chondrocytes upregulate the expressions of β-catenin and LEF-1. β-Catenin, the key protein in the canonical WNT pathway, activates downstream gene expression through binding with lymphoid enhancer factors (LEFs); thus, FAM20B-deficient chondrocytes exhibit functional gain in WNT signaling pathways. In addition, *FAM20B* gene-knockout mice treated with the WNT inhibitor C59 showed improved epiphyseal bone formation and better-shaped metaphysis, partially compensating for postnatal ossification.

#### 4.1.4 FAM198A

The kinase family with the sequence similarity number 198A (FAM198A) is a member of the atypical protein kinases group, secreted through the caveolae biogenesis pathway (42) and localized in the GA and ER (65). Existing studies have shown that the expression of FAM198A in tissues strongly correlates with caveolin-1 and cavin-1 (39–41). It is reported that the FAM198A precursor cannot mature and is secreted out of the cell without cavin-1 because the lack of cavin-1 blocks the FAM198A post-translational process (42). However, cavin-1 is a prognostic biomarker as a target for novel therapeutic strategy development against GBM (88). Therefore, FAM198A may play an essential role in GBM tumorigenesis through the function of cavin-1. It has also been reported that FAM198A mutation is somehow related to drug resistance in non-small-cell lung cancer patients (89). However, the direct effects of FAM198A on different cancer cells require further studies.

#### 4.1.5 Double-edged sword: FAM198B

The kinase family with the sequence similarity number of 198B (FAM198B) is a novel gene in humans with unidentified functions. FAM198B is detected in the nerve cells and epithelium during development, based on various databases. Overexpression of FAM198B increases the risk of drug resistance against cisplatin, topotecan, and paclitaxel and suppresses the proliferation and migration of ovarian cancer cell lines, suggesting a potential role in the treatment of ovarian cancer (90–92). Hsu et al. have reported that the overexpression of FAM198B inhibits the invasion, proliferation, and tumorigenesis of lung adenocarcinoma cells, while the downregulation of FAM198B promotes the occurrence of malignant tumors (93). *FAM198B* was identified as a protective signature gene to predict the prognosis of uterine corpus endometrial carcinoma (94). FAM198B inhibits tumor invasion by downregulating the pERK/MMP1 signaling pathway, while N-glycosylation enhances the FAM198B protein stability. Nearly half of the identified eukaryotic proteins are subjected to N-glycosylation, a ubiquitous post-translational modification (95). The causality has been reported between the alteration of glycosylation and tumor proliferation, invasion, metastasis, angiogenesis, receptor activation, and intracellular or cell–matrix interactions (96–98).

However, recent reports showed that FAM198B can also promote colorectal cancer (CRC) progression (99). The upregulation of FAM198B was found in macrophages and was considered associated with the poor prognosis of CRC. FAM198B promotes M2 macrophage polarization by targeting the SMAD2 pathway, affecting colorectal cancer cell biology in various aspects by regulating the secretion of cytokines, which leads to proliferation, invasion, and migration. This also indicated that targeting FAM198B in macrophages may be an effective strategy to inhibit CRC metastasis.

#### 4.1.6 Tumor promoter: FJX1

Four-jointed box 1 (FJX1), the human homolog of four-jointed box kinase 1, was first identified in 1943 in *Drosophila* genetic studies (100). Subsequent genetic studies of FJX1 have shown that it modulates FAT-Ds binding (101) and interacts genetically with the family of FAT. As FAT3 heterozygotes carry a homozygous mutation in FJX1 and promote FAT signaling (102), FJX1 reduces dendrite extension and branching in hippocampal neurons (103). The abnormal phosphorylation of FJX1 contributes to the duplex kidney phenotype based on its synergistic interaction between FAT4 and FJX1 (104). However, silencing FJX1 does not catalyze the ectodomain phosphorylation of FAT1 (105). FJX1 is vital in the pathogenesis of endometriosis (106). FAT4 inhibits miR-720 expression by repressing the FJX1 plasma levels and endothelial progenitor cells in coronary artery disease patients (107).

Explorations on the function of FJX1 in human cancers have been widely performed. FJX1 is considered a candidate for the regulation of angiogenesis and tumorigenesis-related pathways such as Notch, JAK/STAT, and Hippo-YAP in several human cancers (108–113). Amplification of FJX1 deregulates notch signaling and contributes to the development of oral squamous cell carcinoma (109). *FJX1* is one of the aberrant expression genes involved in the pathogenesis of CRC and functions by regulating proteoglycan expression and cAMP signaling (114). The high expression of FJX1 has negative effects on the overall survival of colon adenocarcinoma (115) and ovarian tumor patients (116). The overexpression of FJX1 is observed at both the mRNA and protein levels in primary nasopharyngeal carcinoma (NPC). It promotes anchorage-independent growth, proliferation, and invasion in NPC cells (108, 117) and could elicit antitumor immune responses in the human peripheral blood monocytes collected from nasopharyngeal carcinoma patients (117). Interestingly, it is feasible that a dual-antigenic peptide vaccine (PV1) comprises both MAGED4B and FJX1 peptides against head and neck squamous cell carcinoma, which is now under investigation in human safety (118).

In addition, various miRNAs interact with FJX1, such as miR-106b-5p; target FJX1 directly, mediating cell proliferation, migration, and invasion in CRC; and decrease with the downregulation of FJX1 (119). The latest research showed that FOXD3-AS1 targets the miR-127-3p/FJX1 axis in melanoma progression (120) and FJX1 facilitates cell proliferation and motility in colon adenocarcinoma by inhibiting miR‐1249 (121, 122).

### 4.2 PKDCC-related kinases

#### 4.2.1 PKDCC

PKDCC (protein kinase domain containing, cytoplasmic, also known as Vlk or sgk493) encodes a protein kinase in a novel family of serine/tyrosine/threonine kinase catalytic domain proteins. It localizes in the Golgi complex, phosphorylates proteins and proteoglycans in the secretory pathway, and regulates the formation of stroma and differentiation of chondrocytes (6, 12, 47, 123–125). The regulation of PKDCC expression and secretion is not yet understood, as well as its physiological function in human cancers, but its function in a variety of diseases has been explored. PKDCC is one of the risk-allele SNP signature genes associated with osteoporosis susceptibility. It may assist in identifying at-risk individuals for the prevention of vertebral fractures (126). Sajan et al. identified the disruption of variants in PKDCC by a biallelic gene in humans. This led to dysmorphic features and rhizomelic shortening of the limbs (127). Furthermore, PKDCC genetically interacts with Gli3 and affects bone development *via* the hedgehog pathway (128). Studies suggested that PKDCC-mediated genetic variation and target protein phosphorylation modulate the axonal pathfinding of retinal ganglion cells (129), lung function measurements (130), atopy (131), and asthma development (132). Maddala et al. suggested that PKDCC maintains the trabecular meshwork cell shape, adhesive properties, and actin stress fiber formation. PKDCC may also play a role in the homeostasis of aqueous humor outflow and intraocular pressure (133). PKDCC regulates Wnt/PCP signaling, a factor impacting the actin cytoskeletal organization and cell adhesion (133, 134). Wnt signaling is involved in the regulation of proliferation and migration of various tumor cells (135, 136), while PKDCC is essential at organogenesis stages for patterning (123, 124, 128). Moreover, PKDCC phosphorylates a broad range of ECM proteins, including collagens, and matrix metalloproteinases (MMPs), i.e., MMP1 and MMP14. Phosphorylation of these proteins potentially controls the protein activities and impacts cell adhesion and migration (12), suggesting a possible functional interaction between PKDCC and cancer.

#### 4.2.2 Kinase-like protein: C3orf58

C3orf58, also named DIPK2A, DIA1, HASF, GoPro49, and PIP49, is an uncharacterized predicted secretory kinase that resides in the GA (137). The human gene *C3orf58* is identified with orthologs in the genome of 10 animal species (137, 138). Several analyses have demonstrated that C3orf58 is widely expressed in the brain tissue, including the hippocampus, olfactory bulb, and cortex (139, 140). As shown in the mass spectrometry proteomic databases, a relatively widespread expression of C3orf58 in the pancreas, adrenal gland, ovary and testis, lung, retina, placenta, and platelets is presented (141, 142). Even so, little is known about its function.

*C3orf58* is one of the four genes most differentially methylated between primary acral lentiginous melanoma and primary non-lentiginous AM or metastatic ALM (143). C3orf58 is a novel paracrine protein that participates in cardiac regeneration and proliferation by stimulating cardiomyocyte division and activating the PI3K–AKT–CDK7 signaling cascade (140). Furthermore, C3orf58 is expressed in cartilaginous mesenchymal tissues and is involved in developmental regulation. The expression is observed the highest in proliferating chondrocytes (137). Moreover, co-localization with beta-coatomer protein is observed, indicating its role in secretory processes (144). The characteristic expression of C3orf58 is presented in dental follicles, confirming its role in secretion and trafficking (144). Moreover, C3orf58 has been characterized as a secreted paracrine factor and ligand for insulin-like growth factor 1 receptor (IGF1R) (145). Evidence has shown that C3orf58 promotes cardiomyocyte regeneration by stimulating proliferation *via* PI3K kinase and by blocking apoptosis *via* PKC-ϵ (140, 146, 147). C3orf58 likely positively regulates CK2 signaling *via* a proline-directed kinase (148). However, the kinase activity of C3orf58 still requires investigation. To fully elucidate the activities of C3orf58 and its signaling network, different cellular systems should be studied.

#### 4.2.3 CXorf36

CXorf36, also named DIA1R or DIPK2B, is regarded as a pseudokinase due to the absence of the predicted conserved active site aspartate (149). The human *CXorf36* gene is X-linked, with its location on the short arm of the X chromosome at site Xp11.3. It has orthologs in at least four vertebrate species (138) and is a highly atypical kinase interfering with another FAM69 protein (149). The *CXorf36* gene is involved in fragile X syndrome, autism, kabuki syndrome, and intellectual disability (150–152).

*CXorf36* is an interchromosomal interaction gene located at the chromosome 8q24 locus that modulates the genetic risk of prostate cancer (153). Therefore, further research on *CXorf36* will pose new ideas on the etiology and regulation of progression in multiple cancers.

#### 4.2.4 Function of undetermined kinase-like proteins: FAM69A, FAM69B, and FAM69C

All three FAM69 proteins (FAM69A, FAM69B, FAM69C) are presented in the ER and act as a putative membrane anchoring molecule (154). The functions of FAM69s are similar to PKDCC and FAM20 novel kinases (10, 11, 47, 64, 65). A genome-wide study has revealed FAM69A mutations related to the increased risk of multiple sclerosis (155), while intronic SNPs of FAM69A are associated with schizophrenia and bipolar disorder (156). Whole exome sequencing identified FAM69A in patient-specific myocytes with altered gene expression in the ophthalmoplegic subphenotype of myasthenia gravis (157). FAM69B was described as pancreatitis-induced protein 49 (PIP49) due to its upregulation function during the acute phase of experimental pancreatic inflammation induced in mice by intraperitoneal injection of caerulein (158). A higher expression of FAM69C is highly sensitive to the second mitochondria-derived activator of caspase mimetics treatment in patients who are suffering from pediatric precursor B-cell acute lymphoblastic leukemia (159).

### 4.3 Tumor promoter: POMK

POMK, also named SGK196, is a type II transmembrane protein that was previously regarded as a “pseudokinase” (3). It has recently been identified to function as a glycosylation-specific kinase (160, 161). It localizes within the ER membrane and functions by adding phosphate to the O-mannose sugar moieties (162, 163).

Mutations of POMK lead to a spectrum of congenital disorders of glycosylation and limb-girdle muscular dystrophies by influencing the biosynthesis of functional alpha-dystroglycan (33, 164–167). POMK mutations cause not only Walker–Warburg syndrome (34) but also a more severe subtype, type C12 (MDDGC12) limb-girdle muscular dystrophy-dystroglycanopathy and type A12 (MDDGA12) congenital muscular dystrophy-dystroglycanopathy with brain and eye anomalies (164, 165, 168, 169). POMK interaction proteins are strongly enriched in membrane proteins and metabolic pathways, demonstrating an important role in developmental and metabolic disorders (167). In 2020, Ci Xu et al. found that N-glycosylated POMK inhibits basal-like breast cancer cell migration, invasion, and metastasis both *in vitro* and *in vivo via* the PI3K/AKT/GSK3β signaling pathway (170).

## 5 Therapeutic strategies targeting SPKKPs

Further understanding the ER stress response and its role in physiology and pathology may reveal a new type of targeted therapy. The UPR is triggered following ER stress response failure. Notably, cancer cells usually show higher basal ER stress than non-tumor cells, and they regulate the UPR to promote tumor growth and survival (23). UPR-dependent extracellular signals can regulate host immune cells by reprogramming cells and regulating secretory pathways. The host’s immune system can be used to fight cancer by mediating the UPR. A variety of pharmacological UPR inhibitors are being developed, and UPR targeting is expected to become a new strategy for disease immunomodulation and immunotherapy (171).

Since most of the molecules involved in intercellular communication are secreted into the extracellular environment through the secretory pathway, SPKKPs play an important role in this process. FAM20C promotes the phosphorylation of secretory proteins under diverse physiological and pathological conditions. These proteins participate in the interaction between the ER and the GA. FAM20C is involved in the supramolecular mechanism directing protein sorting and promoting the appropriate molecule conformation outside the cell (172). It is suggested that the pathological inhibition of FAM20C can inhibit abnormal protein sorting. Existing studies have found two kinds of FAM20C inhibitors for cancer. The potent antiproliferative effects of FL-1607 on triple-negative breast cancer cells were discovered in 2016 including apoptosis induction and cell migration inhibition in MDA-MB-468 cells (173). In addition, 3r featured favorable antiproliferative activity against MDA-MB-231 cells, binding to FAM20C and inducing apoptosis *via* the mitochondrial pathway (174). Moreover, the inhibiting upstream factor S1P reduces the expression of Fam20C. S1P is a Golgi-resident protease and is responsible for the cleavage of the FAM20C propeptide. The S1P inhibitor PF-429242 has been reported to reduce the content of FAM20C in Hela cells (175).

FAM20A potentiates FAM20C kinase activity. FAM20A, a pseudokinase involved in the formation of functional complexes with FAM20C, enhances the phosphorylation of extracellular proteins in the secretory pathway. Therefore, the inhibition of FAM20A may have the same effect on the inhibition of FAM20C. Considering the important role of SPKKPs in tumor progression, further exploration of SPKKP inhibitors is an important direction of antitumor therapy.

**Figure 4:**
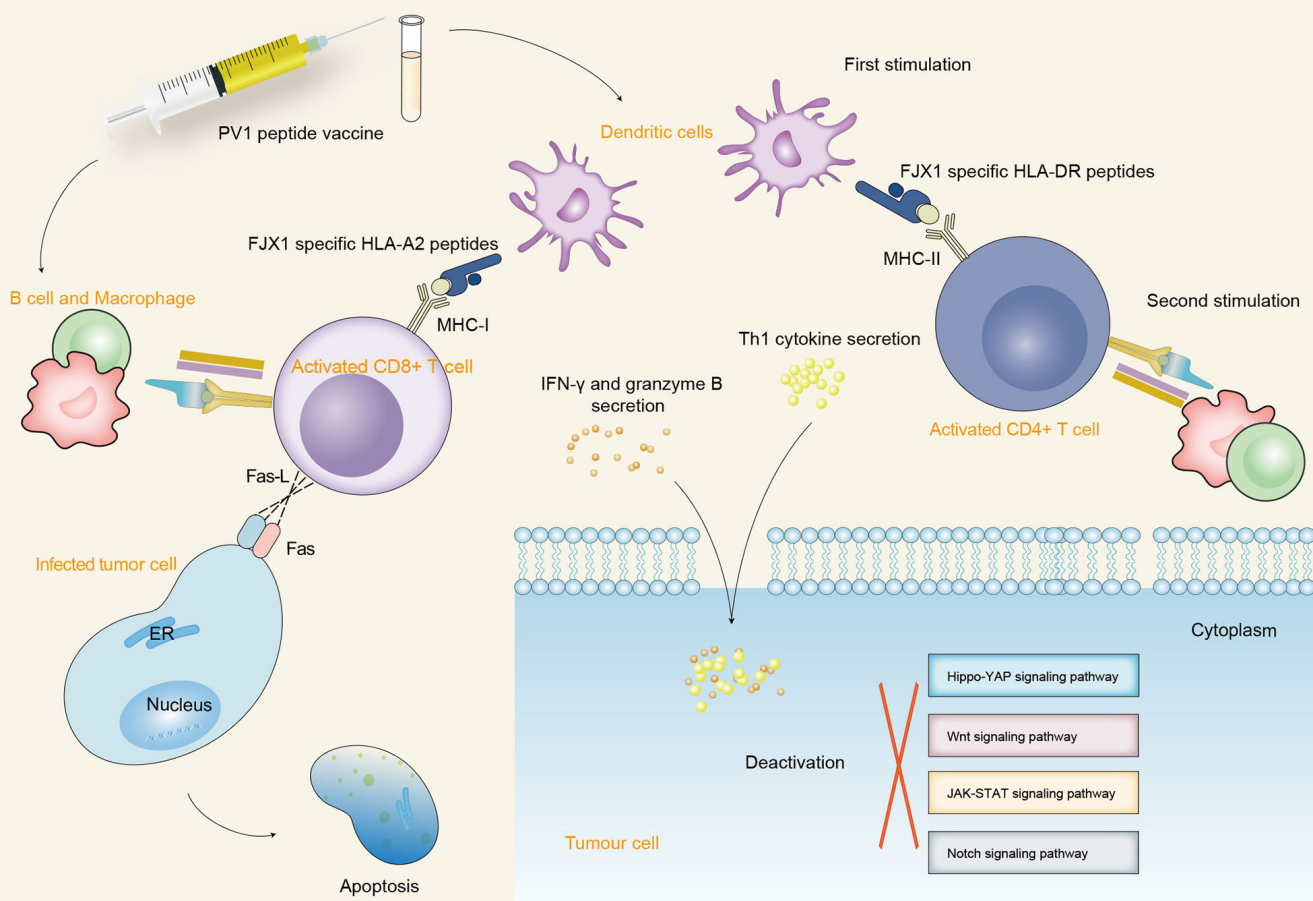
The schematic diagram depicts the immunogenic mechanism of SPKKP-related antigen-specific peptide vaccine in cancers. Dendritic cells participate in the first process of antigen presentation after vaccination, making FJX1-specific HLA-DR and HLA-A2 peptides recognize the specific surface antigen of CD4^+^ T cells and CD8^+^ T cells, respectively. Furthermore, B cells and macrophages activate T cells to produce a long-lasting killing effect and promote the secretion of cytotoxic cytokines. The interaction between Fas-L expressed by CD8^+^ T cells and Fas expressed by target cells promotes the direct apoptosis of tumor cells. The secreted cytotoxic cytokines further inactivate a variety of oncogenic signaling pathways and induce the apoptosis of tumor cells.

The overexpression of FJX1 proteins is observed in both primary and recurrent head and neck squamous cell carcinoma (HNSCC) patients. Recent research showed that a dual-antigenic peptide vaccine (PV1) derived from FJX1 and MAGED4B proteins induces antitumor immune responses *in vitro* (118). Increased infiltration of antigen-specific T cells into the tumor is induced by PV1, which is an important criterion for the success of immunotherapy and is associated with better patient prognosis (176). The cytotoxic cytokines, such as IFN-γ, granzyme B, and Th1, are secreted upon exposure to target cells which express the respective antigen post-PV1 stimulation and further inactivate a variety of oncogenic signaling pathways and induce the apoptosis of tumor cells (118, 176). PV1 inhibits tumor growth and significantly improves the prognosis when used alone or in combination with anti-PD1 (176). HNSCC patients are more responsive to PV1 stimulation, as it not only induces tumor-specific immune responses but also promotes the establishment of immunological memory. This demonstrated that PV1 may be an effective vaccine for the treatment of HNSCC and other cancer patients with FJX1 overexpression (**Figure 4**).

## 6 Conclusions and perspectives

Various evidence showed that carcinogenesis is related to the overproduction or delayed clearance of protein complexes. This is a new focus for new drug development. The summary of the function and molecular mechanism of kinase or kinase-like protein in the secretory pathway provides scientists with a direction for the latest drug target research. It is also important to understand the effects of SPKKPs in various organelles within the secretory pathway, ER stress, and UPR. The purpose is to increase the accumulation of immature proteins, clear abnormal unfolded proteins, and enhance cell viability. In this perspective, a variety of drugs should be studied to identify potential targets for abnormal ER stress or abnormal UPR to promote protein homeostasis and antitumor effects. In this review, we described 13 kinases or kinase-like proteins in the secretory pathway, all of which are specifically presented in the GA and ER. Some of these are secreted into the extracellular space to take effect functionally. Some play key roles in cell biology and the cell mutations may lead to human diseases. Understanding the mechanisms of kinase regulation and the recognition of their substrates facilitates a better understanding of the occurrence and development of human diseases.

**Figure 5:**
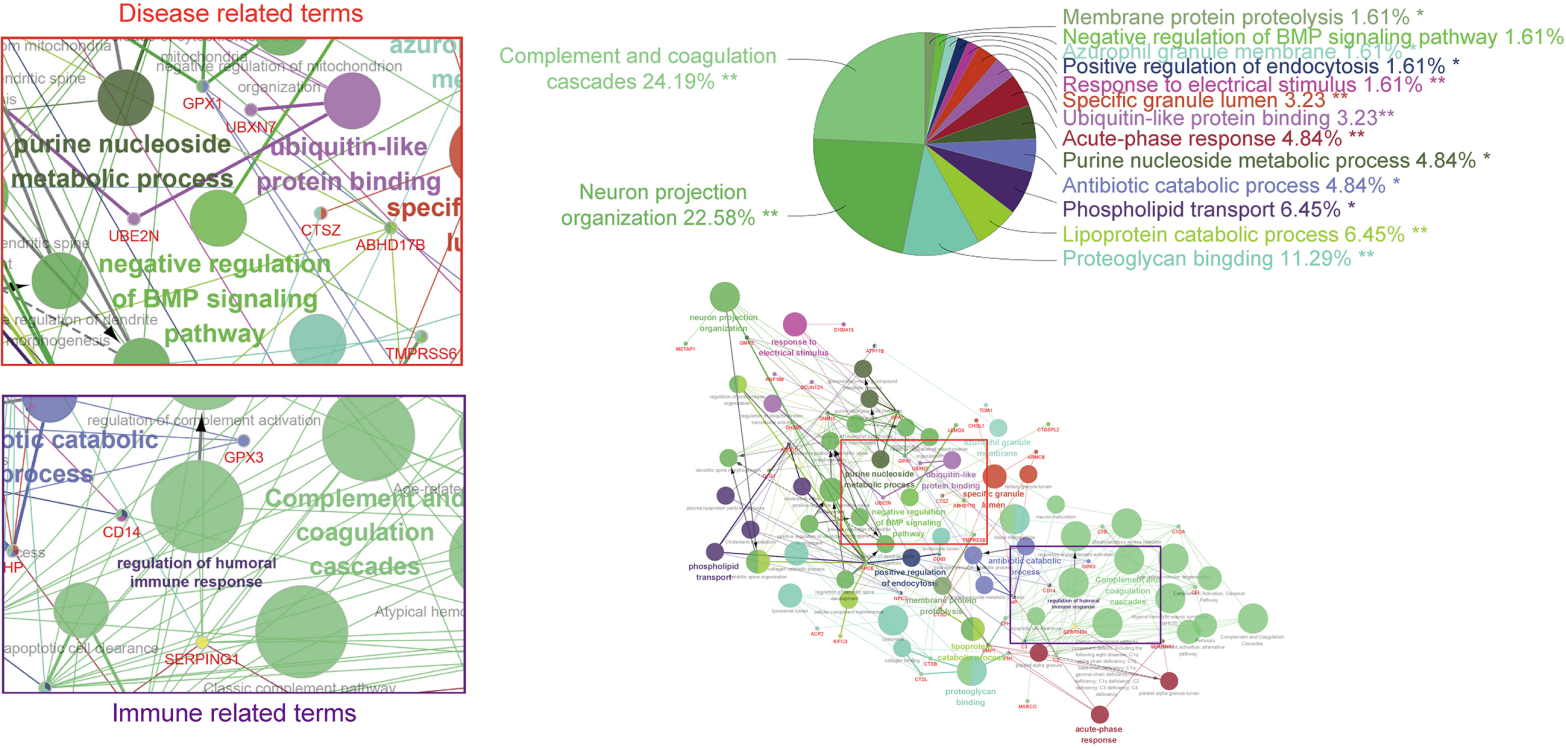
GO and KEGG analysis based on co-regulated genes with the SPKKP signature score in pan-cancer. Each node represents a gene ontology term, while the edges are quantitative representations of shared gene membership. The different colors of the nodes represent the different groups generated. The pie chart represents the ClueGo pathway groups by the percentage of gene ontology (GO) terms contained.

Relevant bioinformatic analyses reported that some of these proteins, including FAM20C, FJX1, or POMK, represent a tumor-promoting phenotype. However, the mechanism of the impact on tumorigenesis remains obscure. Functional analysis data showed that SPKKP-related genes are mainly enriched in disease- and immune-related terms (**Figure 5**). In-depth structural and functional studies are necessary to better feature the characteristics of related phenotypes. Canonical treatments (protease and/or RNA polymerase inhibitors) accompanied by drugs targeting secretory pathway components can achieve synergistic effects since cancer cell propagation is dependent on the efficiency of secretory organelles. Given the strong interconnections of tumorigenesis and innate immunity, increasing attention should be paid to the secretory pathway kinases. The reduction of ER stress and enhancement of protein folding promote chaperone production and secretion, establishing an immunosuppressive microenvironment for tumor growth. ER-associated degradation is a critical pathway in the limitation of protein condensation and aggregation in the early secretory compartment (177). Therefore, studies on the interconnections of SPKKPs, cancer cells, and immune cells are required. The identification of new secretory pathway-related targets facilitates the development of antitumor therapy in a more specific manner and also sketches an outline for future studies on tumorigenesis.

## Author contributions

SD and SG conceived and designed the study. XCh, XR and XC performed the bioinformatic analysis and drew the plots. SD, CZ and SG drafted the manuscript. SD and CZ obtained funding for the study. All authors made a significant contribution to the work reported, either in the conception, study design, execution, acquisition of data, analysis, and interpretation, or in all the above mentioned areas; took part in drafting, revising, and critically reviewing the article; gave final approval of the version to be published; have agreed on the journal to which the article has been submitted; and agreed to be accountable for all aspects of the work.

## Supplementary material

Supplementary Table 1 Targets and cancer references of SPKKPs.
