# Supplementary material for: Regulation of secretory pathway kinase or kinase-like proteins in human cancers
Source: Front Immunol. 2023 Feb 7;14:942849. doi: 10.3389/fimmu.2023.942849 (PMC9941534; doi:10.3389/fimmu.2023.942849)
Supplement: Supplementary Table 1 — Targets and cancer references of SPKKPs. [file Table_1.pdf]

Targets and cancer references of SPKKPs

Gene name	Number of transcript variants	Targets or interacted genes	Function	Cancer and reference
**FAM198B**	3	ERK1,	Tumor suppressor	Lung adenocarcinoma (1)<br>Acute aortic dissection (2)<br>Head and neck squamous cell carcinoma (3)<br>Ovarian cancer (4)
**FAM198A**	-	CAVIN16, EGFR5	Not reported	Non-small-cell lung cancer chemotherapy resistance (5, 6)
**FJX1**	-	MiR-106b-5p7, PVT17, miR-127-3p9, FOXD39, MicroRNA-124910, HLA-A212	Tumor promoter	Colorectal cancer (7, 8)<br>Melanoma (9)<br>Colon adenocarcinoma (10)<br>Nasopharyngeal carcinoma (11, 12)<br>Head and neck cancer (13)<br>Head and neck squamous cell carcinoma (14)<br>Endometriosis (15)<br>Coronary artery disease (16)<br>Non-small cell lung cancer (17)<br>Cystic kidney disease(18)<br>Oral squamous cell carcinoma (19)
**FAM20B**	3	Not reported	Not reported	Chondrosarcoma and postnatal ossification defects (20)
**FAM20A**	3	Not reported	Not reported	Amelogenesis imperfecta and gingival hyperplasia syndrome (21)<br>Enamel renal syndrome (22-24)<br>Periodontal disease (25)<br>Hematopoiesis (26)<br>Parathyroid carcinoma (27)
**FAM20C**	-	FGF23, PCSK9, IGFBP3, PCSK7, OPN, BMP4	Tumor promoter	Glioma (28, 29)<br>Breast cancer (30-32)<br>Testicular cancer(33)
**CXorf36**	2	Not reported	Tumor promoter	Fragile X syndrome(34, 35)<br>X-linked mental retardation (XLMR) (36)<br>Kabuki syndrome (37)<br>Prostate cancer (38)
**FAM69C**	-	Not reported	Not reported	Not reported
**FAM69B**	-	Not reported	Not reported	Pancreatic inflammation (39)
**FAM69A**	5	Not reported	Not reported	Multiple sclerosis (40)

**PKDCC**	-	Not reported	Not reported	Osteoporosis (41)
**POMK**	2	NRG137, α-DG36	Tumor promoter	Breast cancer (42)<br>Colorectal cancer (43)
**C3orf58**	3	Not reported	Not reported	Cardiomyocyte cell-cycle progression (44)<br>Acral melanomas(45)<br>Cartilaginous mesenchymal tissues(46)

References

1. Hsu CY, Chang GC, Chen YJ, Hsu YC, Hsiao YJ, Su KY et al. FAM198B Is Associated with Prolonged Survival and Inhibits Metastasis in Lung Adenocarcinoma via Blockage of ERK-Mediated MMP-1 Expression. *Clin Cancer Res* (2018) 24(4), 916-926. doi:10.1158/1078-0432.CCR-17-1347
2. Wang T, He X, Liu X, Liu Y, Zhang W, Huang Q et al. Weighted Gene Co-expression Network Analysis Identifies FKBP11 as a Key Regulator in Acute Aortic Dissection through a NF-kB Dependent Pathway. *Front Physiol* (2017) 8, 1010. doi:10.3389/fphys.2017.01010
3. Zhang Z, Liu R, Jin R, Fan Y, Li T, Shuai Y et al. Integrating Clinical and Genetic Analysis of Perineural Invasion in Head and Neck Squamous Cell Carcinoma. *Front Oncol* (2019) 9, 434. doi:10.3389/fonc.2019.00434
4. Swierczewska M, Klejewski A, Brazert M, Kazmierczak D, Izycki D, Nowicki M et al. New and Old Genes Associated with Primary and Established Responses to Paclitaxel Treatment in Ovarian Cancer Cell Lines. *Molecules* (2018) 23(4), 891. doi:10.3390/molecules23040891
5. van der Wekken AJ, Kuiper JL, Saber A, Terpstra MM, Wei J, Hiltermann TJN et al. Overall survival in EGFR mutated non-small-cell lung cancer patients treated with afatinib after EGFR TKI and resistant mechanisms upon disease progression. *PLoS One* (2017) 12(8), e0182885. doi:10.1371/journal.pone.0182885
6. Wei Z, Liu T, Lei J, Wu Y, Wang S and Liao K. Fam198a, a member of secreted kinase, secrets through caveolae biogenesis pathway. *Acta Biochim Biophys Sin (Shanghai)* (2018) 50(10), 968-975. doi:10.1093/abbs/gmy105
7. Liu F, Wu R, Guan L and Tang X. Knockdown of PVT1 Suppresses Colorectal Cancer Progression by Regulating MiR-106b-5p/FJX1 Axis. *Cancer Manag Res* (2020) 12, 8773-8785. doi:10.2147/CMAR.S260537
8. Al-Greene NT, Means AL, Lu P, Jiang A, Schmidt CR, Chakravarthy AB et al. Four jointed box 1 promotes angiogenesis and is associated with poor patient survival in colorectal carcinoma. *PLoS One* (2013) 8(7), e69660. doi:10.1371/journal.pone.0069660
9. Wan N, Yang W, Cheng H and Wang J. FOXD3-AS1 Contributes to the Progression of Melanoma Via miR-127-3p/FJX1 Axis. *Cancer Biother Radiopharm* (2020) 35(8), 596-604. doi:10.1089/cbr.2019.3093
10. Dang W and Zhu Z. MicroRNA-1249 targets four-jointed box kinase 1 and reduces cell proliferation, migration and invasion of colon adenocarcinoma. *J Gene Med* (2020) 22(7), e3183. doi:10.1002/jgm.3183
11. Chai SJ, Ahmad Zabidi MM, Gan SP, Rajadurai P, Lim PVH, Ng CC et al. An Oncogenic Role for Four-Jointed Box 1 (FJX1) in Nasopharyngeal Carcinoma. *Dis Markers* (2019) 2019, 3857853.

doi:10.1155/2019/3857853
12. Chai SJ, Yap YY, Foo YC, Yap LF, Ponniah S, Teo SH et al. Identification of Four-Jointed Box 1 (FJX1)-Specific Peptides for Immunotherapy of Nasopharyngeal Carcinoma. *PLoS One* (2015) 10(11), e0130464. doi:10.1371/journal.pone.0130464
13. Chai SJ, Fong SCY, Gan CP, Pua KC, Lim PVH, Lau SH et al. In vitro evaluation of dual-antigenic PV1 peptide vaccine in head and neck cancer patients. *Hum Vaccin Immunother* (2019) 15(1), 167-178. doi:10.1080/21645515.2018.1520584
14. Jarvinen AK, Autio R, Kilpinen S, Saarela M, Leivo I, Grenman R et al. High-resolution copy number and gene expression microarray analyses of head and neck squamous cell carcinoma cell lines of tongue and larynx. *Genes Chromosomes Cancer* (2008) 47(6), 500-9. doi:10.1002/gcc.20551
15. Chang HJ, Yoo JY, Kim TH, Fazleabas AT, Young SL, Lessey BA et al. Overexpression of Four Joint Box-1 Protein (FJX1) in Eutopic Endometrium From Women With Endometriosis. *Reprod Sci* (2018) 25(2), 207-213. doi:10.1177/1933719117716780
16. Wang HW, Huang TS, Lo HH, Huang PH, Lin CC, Chang SJ et al. Deficiency of the microRNA-31-microRNA-720 pathway in the plasma and endothelial progenitor cells from patients with coronary artery disease. *Arterioscler Thromb Vasc Biol* (2014) 34(4), 857-69. doi:10.1161/ATVBAHA.113.303001
17. Chang JW, Wei NC, Su HJ, Huang JL, Chen TC, Wu YC et al. Comparison of genomic signatures of non-small cell lung cancer recurrence between two microarray platforms. *Anticancer Res* (2012) 32(4), 1259-65.
18. Saburi S, Hester I, Fischer E, Pontoglio M, Eremina V, Gessler M et al. Loss of Fat4 disrupts PCP signaling and oriented cell division and leads to cystic kidney disease. *Nature Genetics* (2008) 40, 1010-1015. doi:10.1038/ng.179
19. Snijders AM, Schmidt BL, Fridlyand J, Dekker N, Pinkel D, Jordan RC et al. Rare amplicons implicate frequent deregulation of cell fate specification pathways in oral squamous cell carcinoma. *Oncogene* (2005) 24(26), 4232-42. doi:10.1038/sj.onc.1208601
20. Ma P, Yan W, Tian Y, Wang J, Feng JQ, Qin C et al. Inactivation of Fam20B in Joint Cartilage Leads to Chondrosarcoma and Postnatal Ossification Defects. *Sci Rep* (2016) 6, 29814. doi:10.1038/srep29814
21. O'Sullivan J, Bitu CC, Daly SB, Urquhart JE, Barron MJ, Bhaskar SS et al. Whole-Exome sequencing identifies FAM20A mutations as a cause of amelogenesis imperfecta and gingival hyperplasia syndrome. *Am J Hum Genet* (2011) 88(5), 616-20. doi:10.1016/j.ajhg.2011.04.005
22. Vogel P, Hansen GM, Read RW, Vance RB, Thiel M, Liu J et al. Amelogenesis imperfecta and other biomineralization defects in Fam20a and Fam20c null mice. *Vet Pathol* (2012) 49(6), 998-1017. doi:10.1177/0300985812453177
23. Jaureguiberry G, De la Dure-Molla M, Parry D, Quentric M, Himmerkus N, Koike T et al. Nephrocalcinosis (enamel renal syndrome) caused by autosomal recessive FAM20A mutations. *Nephron Physiol* (2012) 122(1-2), 1-6. doi:10.1159/000349989
24. Wang SK, Aref P, Hu Y, Milkovich RN, Simmer JP, El-Khateeb M et al. FAM20A mutations can cause enamel-renal syndrome (ERS). *PLoS Genet* (2013) 9(2), e1003302. doi:10.1371/journal.pgen.1003302
25. Kantaputra PN, Bongkochwilawan C, Lubinsky M, Pata S, Kaewgahya M, Tong HJ et al. Periodontal disease and FAM20A mutations. *J Hum Genet* (2017) 62(7), 679-686.

doi:10.1038/jhg.2017.26
26. Nalbant D, Youn H, Nalbant SI, Sharma S, Cobos E, Beale EG et al. FAM20: an evolutionarily conserved family of secreted proteins expressed in hematopoietic cells. *BMC Genomics* (2005) 6, 11. doi:10.1186/1471-2164-6-11
27. Hu Y, Zhang X, Wang O, Bi Y, Xing X, Cui M et al. The genomic profile of parathyroid carcinoma based on whole-genome sequencing. *Int J Cancer* (2020) 147(9), 2446-2457. doi:10.1002/ijc.33166
28. Du S, Guan S, Zhu C, Guo Q, Cao J, Guan G et al. Secretory Pathway Kinase FAM20C, a Marker for Glioma Invasion and Malignancy, Predicts Poor Prognosis of Glioma. *Onco Targets Ther* (2020) 13, 11755-11768. doi:10.2147/OTT.S275452
29. Feng J, Zhou J, Zhao L, Wang X, Ma D, Xu B et al. Fam20C Overexpression Predicts Poor Outcomes and is a Diagnostic Biomarker in Lower-Grade Glioma. *Front Genet* (2021) 12(1664-8021 (Print)), 757014. doi:10.3389/fgene.2021.757014
30. Zuo H, Yang D and Wan Y. Fam20C Regulates Bone Resorption and Breast Cancer Bone Metastasis through Osteopontin and BMP4. *Cancer Res* (2021) 81(20), 5242-5254. doi:10.1158/0008-5472.CAN-20-3328
31. Zhao R, Fu L, Yuan Z, Liu Y, Zhang K, Chen Y et al. Discovery of a novel small-molecule inhibitor of Fam20C that induces apoptosis and inhibits migration in triple negative breast cancer. *Eur J Med Chem* (2021) 210, 113088. doi:10.1016/j.ejmech.2020.113088
32. Tagliabracci VS, Wiley SE, Guo X, Kinch LN, Durrant E, Wen J et al. A Single Kinase Generates the Majority of the Secreted Phosphoproteome. *Cell* (2015) 161(7), 1619-32. doi:10.1016/j.cell.2015.05.028
33. Trendowski MR, Wheeler HE, El-Charif O, Feldman DR, Hamilton RJ, Vaughn DJ et al. Clinical and Genome-Wide Analysis of Multiple Severe Cisplatin-Induced Neurotoxicities in Adult-Onset Cancer Survivors. *Clin Cancer Res* (2020) 26(24), 6550-6558. doi:10.1158/1078-0432.CCR-20-2682
34. Jensen LR, Lenzner S, Moser B, Freude K, Tzschach A, Wei C et al. X-linked mental retardation: a comprehensive molecular screen of 47 candidate genes from a 7.4 Mb interval in Xp11. *Eur J Hum Genet* (2007) 15(1), 68-75. doi:10.1038/sj.ejhg.5201714
35. Tarpey PS, Smith R, Pleasance E, Whibley A, Edkins S, Hardy C et al. A systematic, large-scale resequencing screen of X-chromosome coding exons in mental retardation. *Nat Genet* (2009) 41(5), 535-43. doi:10.1038/ng.367
36. Thiselton DL, McDowall J, Brandau O, Ramser J, d'Esposito F, Bhattacharya SS et al. An integrated, functionally annotated gene map of the DXS8026-ELK1 interval on human Xp11.3-Xp11.23: potential hotspot for neurogenetic disorders. *Genomics* (2002) 79(4), 560-72. doi:10.1006/geno.2002.6733
37. Lederer D, Grisart B, Digilio MC, Benoit V, Crespin M, Ghariani SC et al. Deletion of KDM6A, a histone demethylase interacting with MLL2, in three patients with Kabuki syndrome. *Am J Hum Genet* (2012) 90(1), 119-24. doi:10.1016/j.ajhg.2011.11.021
38. Du M, Yuan T, Schilter KF, Dittmar RL, Mackinnon A, Huang X et al. Prostate cancer risk locus at 8q24 as a regulatory hub by physical interactions with multiple genomic loci across the genome. *Hum Mol Genet* (2015) 24(1), 154-66. doi:10.1093/hmg/ddu426
39. Samir AA, Ropolo A, Grasso D, Tomasini R, Dagorn JC, Dusetti N et al. Cloning and expression of the mouse PIP49 (Pancreatitis Induced Protein 49) mRNA which encodes a new putative

transmembrane protein activated in the pancreas with acute pancreatitis. *Mol Cell Biol Res Commun* (2000) 4(3), 188-93. doi:10.1006/mcbr.2000.0277
40. Alcina A, Fernandez O, Gonzalez JR, Catala-Rabasa A, Fedetz M, Ndagire D et al. Tag-SNP analysis of the GFI1-EVI5-RPL5-FAM69 risk locus for multiple sclerosis. *Eur J Hum Genet* (2010) 18(7), 827-31. doi:10.1038/ejhg.2009.240
41. Zhou H, Mori S, Ishizaki T, Takahashi A, Matsuda K, Koretsune Y et al. Genetic risk score based on the prevalence of vertebral fracture in Japanese women with osteoporosis. *Bone Rep* (2016) 5, 168-172. doi:10.1016/j.bonr.2016.07.001
42. Xu C, Zhang M, Bian L, Li Y, Yao Y and Li D. N-glycosylated SGK196 suppresses the metastasis of basal-like breast cancer cells. *Oncogenesis* (2020) 9(1), 4. doi:10.1038/s41389-019-0188-1
43. Cadranel J, Liu SV, Duruisseaux M, Branden E, Goto Y, Weinberg BA et al. Therapeutic Potential of Afatinib in NRG1 Fusion-Driven Solid Tumors: A Case Series. *Oncologist* (2021) 26(1), 7-16. doi:10.1634/theoncologist.2020-0379
44. Beigi F, Schmeckpeper J, Pow-Anpongkul P, Payne JA, Zhang L, Zhang Z et al. C3orf58, a novel paracrine protein, stimulates cardiomyocyte cell-cycle progression through the PI3K-AKT-CDK7 pathway. *Circ Res* (2013) 113(4), 372-80. doi:10.1161/CIRCRESAHA.113.301075
45. Pradhan D, Jour G, Milton D, Vasudevaraja V, Tetzlaff MT, Nagarajan P et al. Aberrant DNA Methylation Predicts Melanoma-Specific Survival in Patients with Acral Melanoma. *Cancers (Basel)* (2019) 11(12), 2031. doi:10.3390/cancers11122031
46. Takatalo M, Jarvinen E, Laitinen S, Thesleff I and Ronnholm R. Expression of the novel Golgi protein GoPro49 is developmentally regulated during mesenchymal differentiation. *Dev Dyn* (2008) 237(8), 2243-55. doi:10.1002/dvdy.21646
